# Measuring the time costs of exercise: a proposed measuring method and a pilot study

**DOI:** 10.1186/1478-7547-8-9

**Published:** 2010-05-11

**Authors:** Lars Axel Hagberg, Lars Lindholm

**Affiliations:** 1 Department of Social Medicine and Public Health, and Centre for Health Care Science, Örebro County Council, Örebro, Sweden; 2 Department of Public Health and Clinical Medicine, Umeå University, Umeå, Sweden

## Abstract

**Background:**

The cost of time spent on exercise is an important factor in societal-perspective health economic analyses of interventions aimed at promoting physical activity. However, there are no existing measuring methods for estimating time costs. The aim of this article is to describe a way to measure the costs of time spent on physical activity. We propose a model for measuring these time costs, and present the results of a pilot study applying this model to different groups of exercisers.

**Methods:**

We began this investigation by developing a model for measuring the time spent on exercise, based on the most important theoretical frameworks for valuing time. In the model, the value of utility in anticipation (expected health benefits) of performing exercise is expressed in terms of health-related quality of life. With this approach, the cost of the time spent on exercise is defined as the value of utility in use of leisure activity forgone minus the value of utility in use of exercise. Utility in use for exercise is valued in comparison with utility in use for leisure activity forgone and utility in use for work.

To put the model into practice, we developed a questionnaire with the aim of investigating the valuations made by exercisers, and applied this questionnaire among more experienced and less experienced exercisers.

**Results:**

Less experienced exercisers valued the time spent on exercise as being equal to 26% of net wages, while more experienced exercisers valued this time at 7% of net wages (p < 0.001). The higher time costs seen among the less experienced exercisers correlated to a less positive experience of exercise and a more positive experience of the lost leisure activity. There was a significant inverse correlation between the costs of time spent on exercise, and the frequency and duration of regular exercise.

**Conclusion:**

The time spent on exercise is an important factor in interventions aimed at promoting physical activity, and should be taken into consideration in cost-effectiveness analyses. The proposed model for measuring the costs of the time spent on exercise seems to be a better method than the previously-used assumptions of time costs.

## Background

Physical activity prevents a number of diseases [1,2], and has an impact on health-related quality of life [3]. The society have a number of inputs with aim to promote physical; for example, physical education in schools is one way to create a healthy and physically active way of life. Furthermore, campaigns with the aim of promoting physical activity are common in society, and physical activity is often used in health care as both treatment and prevention.

Cost-effectiveness evaluations can be performed from different perspectives, but in general a societal perspective is recommended [4-6]. From this perspective, the time spent on exercise is usually one of the greatest inputs in any intervention to promote physical activity. Guidelines for cost-effectiveness analysis seldom discuss the costs of patients' leisure time, though they often include the time spent on informal care and volunteer time, which both have a number of similarities with patients' leisure time. Different sets of guidelines do not always agree on whether informal care should be included in analyses, but volunteer time should always be identified and included unless deemed to be minimal [4]. Hence, time costs of exercise should be considered, or at least discussed, in economic evaluations of interventions aimed at promoting physical activity. A review of articles concerning the cost-effectiveness of physical activity promotion in health care identified 26 articles. Six of these articles included the costs of exercise time; however, all six simply made assumptions about the time costs, rather than soliciting valuations from the participants [7]. Hatziandreu [8] was a trend-setter in this field, using the assumption that exercise time should be valued at full wages for those who dislike exercise, at half wages for those who are neutral, and at no cost for those who like exercise.

The aim of this article is to describe a way of valuing the time spent on physical activity. This aim is achieved by

- proposing a model for measuring the costs of the time spent on exercise, and

- testing the model in different groups of exercisers.

## Methods

### Theoretical framework

Theoretical frameworks for valuing time have mostly been developed for estimating the costs of travel time, but should also be helpful for valuing exercise time.

Time is usually regarded as an economic resource which is possessed by all individuals in the same fixed quantity. Individuals may allocate their time to different activities in different quantities in such a way that each time allocation will have its own consequences for the individual's budget and utility level. Individuals are assumed to choose in such a way that their personal utility will be maximized. Unlike other utility, such as money, time-dependent utility cannot be stored, and as such can only be transferred between those activities which may be interchanged at a particular moment.

Becker was an early pioneer of theories of valuing time. He formulated a model based on incorporation of non-working time where utility depends on the consumption of basic commodities, which requires both market goods and time [9]. These commodities are then combined in a way which maximizes utility. The theory implies that a reallocation of time implies a simultaneous reallocation of goods and commodities (like household work); thus these three decisions are all connected. Becker also pointed out that time can be converted into goods by using less time for consumption and more for work.

These theories were further developed by De Serpa in the seventies [10]. De Serpa considered time to be a resource, and hence a commodity. He also included working time in his model, as well as budget and time constraints. In his theory, the value of time as a resource corresponds to the marginal rate of substitution between time and income, and this rate indicates the monetary value of having an additional unit of time (i.e. saving an unit of time). According to De Serpa, in activities, such as leisure activities, where constraint does not come into play (when an individual devotes more time than the minimum required for the consumption of a good), the dual variable will be zero and therefore the value of time saving in the consumption of the activity will also be zero. This indicates that the utility derived from the time used in the consumption of this good is equal to the value of time as a resource.

Several improvements in valuing time have been made since the work done by De Serpa; in terms of valuing exercise time, the work of Jara-Diaz is probably the most important. He emphasized that the basic source of utility is the time spent on different activities [11]. In this model, it is assumed that goods are not only needed to perform the different activities, but are also the main source of expenses. The time assigned to each activity is related to that assigned to other activities in two ways: through direct dependence among time spent on different activities (i.e. the amount of time spent on an activity influences the amount of time spent on any other activity), and through the shared use of goods. Hence, time is the main utility source, and goods must be considered not only as an end, but also as means to an end.

It is obvious that there is good theoretical support for the assumption that the value of time is equal to the value of the utility that an individual receives from an activity, and that the difference in value between two activities can be regarded as the difference in individual utility [5].

Utility can be divided into two parts; utility during the performance of the activity (utility in use, or process utility) and utility after the activity is performed (utility in anticipation, or outcome utility) [12]. This is an important distinction when it comes to time spent on exercise, which is mostly motivated by both enjoyment (utility in use) and better health (utility in anticipation).

In economic evaluations (cost-utility analysis), utility in anticipation of exercise should be expressed in terms such as quality-adjusted life years (QALY), as should the possible losses of utility in anticipation for the activity forgone in favor of exercise [13]. However, utility in use cannot be captured using QALY measures, because QALY are not particularly sensitive to enjoyment), and the only possible solution is to monetarize it [13].

**Figure 1:**
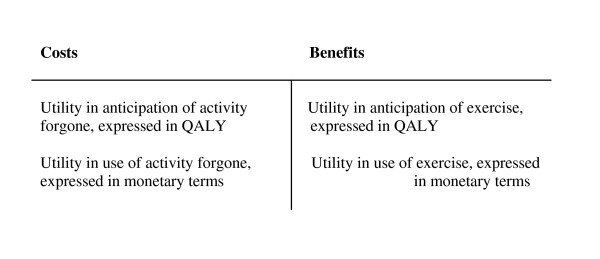
**Costs and benefits of time spent on exercise**.

When using these theories for measuring the costs of exercise time, the opportunity cost of exercise time and the utility of the activity are important. Both opportunity costs (the activity forgone when exercising) and the value of exercise should be divided into utility in use and utility in anticipation. The costs and benefits for an individual of the time spent on exercise can be expressed as shown in Figure 1.

In an economic evaluation of exercise, with health gains expressed in QALY, the monetarized cost of time is only a matter of utility in use. The following can be stated:

#### The monetarized cost of time spent on exercise is the value of utility in use of leisure activity forgone minus the value of utility in use of exercise

**Figure 2:**
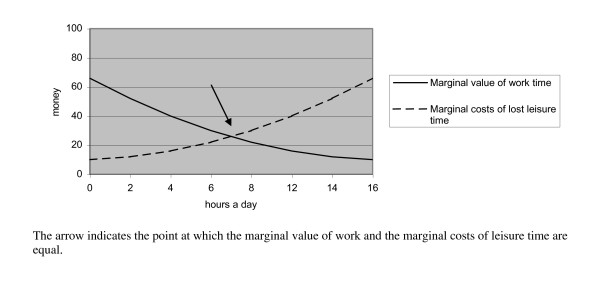
**Marginal value of work and marginal costs of lost leisure time**.

The value of the leisure activity forgone is the opportunity cost of time, and the wages received for work are central to this valuation. In the context of opportunity costs, marginal value is key to this line of thinking. The marginal value of work is assumed to decrease; in principle, the first hour's work of the day is the most valuable, and may be necessary for survival, while the last hour of working is least valuable and may only increase the possibility of extended consumption. Similarly, the marginal value of lost leisure time can be assumed to increase with working time, since if an individual sleeps for about 8 hours then the constraint is about 16 hours to share between work and leisure. The first hour of lost leisure time in a day may not cost an individual much; perhaps only the lost value of extended television-watching. However, the loss of the last hour is more significant; this hour will be very valuable, being potentially needed for activities such as eating or taking care of one's family. Figure 2 illustrates this pattern of decreasing marginal value of work and increasing marginal costs of lost leisure time when working time is expended.

The individual is assumed to work up to the point at which the marginal value of work is equal to the marginal cost of lost leisure time [14] (see the arrow in Figure 2). In general, activities lost due to increased exercise are assumed to be the least valuable leisure activities, and hence their value is equal to the marginal value of work and so can be represented by net wages [14].

Work is not only a matter of wages and utility in anticipation. In fact, many people enjoy their work, and so work also has value of utility in use. The wages received in return for work can be assumed to represent the difference between utility in use of lost leisure activity and utility in use of work.

### Measurement method

Our method of measuring costs for time spent on exercise is thus based on the assumption that work (on the margin) represents a loss of utility in use compared to utility of use of the least valued leisure hour, and that this difference is compensated by net wage. The method requires two simplifying assumptions; that work does not include any utility in anticipation, and that the leisure activity forgone by taking exercise contains only utility in use.

These two anchoring points -- utility in use of work at the margin, and utility in use of the leisure activity forgone in favor of exercise -- can be used as a yardstick for the measurement of the value of utility in use of exercise. The gap between these two points is equivalent to net wage.

'Utility in use' is a technical term, which will not be correctly understood by people in general. Thus, one crucial issue was to find an easily understood term to cover utility in use, and only utility in use. Two notions were considered. 'Enjoyment' may cover many aspects of utility in use, but it does not include aspects such as pain reduction and well-being. 'Positive experience of time' may cover enjoyment, but also includes other aspects of utility in use. One important criterion was that the chosen term must not contain any utility in anticipation. Positive experience of time was decided to best represent utility in use, and was assumed to not contain any utility in anticipation.

**Figure 3:**
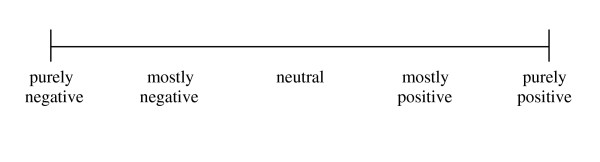
**Experience of activity graphic rating scale**.

The respondents were asked to evaluate and mark their experiences of paid work, lost leisure activity, and exercise on a graphical rating scale. See Figure 3. It was stressed that the lost leisure activity had to be the least valuable enjoyment activity, and that only the experiences while the activity was in progress should be judged. The options on the scale were assumed to be equidistant.

The questionnaire used in the investigation consisted of three main parts: 1) identification of the leisure activity forgone, 2) rating of the experience of time spent on exercise, work, and the leisure activity forgone, and 3) collection of data regarding exercise habits and other background variables.

In measuring costs of the time spent on exercise, the following interpretations were made:

1. When the experience of exercise was graded higher than or at the same level as the leisure activity forgone, the value of utility in use of exercise was the same as for the leisure activity forgone.

2. When the experience of exercise was graded lower than the activity forgone, and graded lower than or at the same level as work, the value of utility in use of exercise was zero.

3. When the experience of exercise was graded in between the experience of work and that of the leisure activity forgone, the value of utility in use of exercise was determined by its position on the scale relative to the positions of work and leisure activity.

As an example, assume that, on a scale of 0-100, an exerciser valued the experience of work at 30 and the experience of the leisure activity forgone at 70. If this individual then valued the experience of exercise at 30, this would imply a claim of net wages as compensation for reaching an utility level equivalent to 70, while a valuation of 70 would imply no claim of net wages, and a valuation of 50 would lie halfway between these points and thus represent a claim of half net wages.

### Material

The investigation was performed in two different groups:

- more experienced exercisers (inclusion criteria: had exercised at least once a week for two years or more, and aged between 20 and 65)

- less experienced exercisers (inclusion criteria: had not exercised at least once a week for two years or more, and aged between 20 and 65)

These groups were chosen on the hypothesis that their time costs would differ, in order to indicate the costs of time both at the beginning of an intervention and in the long run. Invitations to participate were extended to the first 82 individuals who were encountered at a exercise centre, and the first 123 individuals who received exercise on prescription in two county councils.

The characteristics of the participants are presented in Table 1.

**Table 1:** Characteristics of the participants

	n	Age in years	Proportion female	Proportion in work
Experienced exercisers	164	50	87%	67%
Inexperienced exercisers	41	45	90%	68%

### Statistical analysis

Differences between more and less experienced exercisers in cost of time and ratings of the experience of leisure time forgone, exercise, and work were analyzed using the independent t-test.

Spearman's rank correlation was used to examine the association between costs of time spent on exercise and experience of leisure activity forgone, exercise, and work, as well as frequency and duration of exercise.

## Results

When asked which activities they would give up in order to increase their exercise time, 6% of the group of more experienced exercisers stated that they would forego work, 45% that they would forego housework, and 49% that they would forego enjoyable leisure activities. In the group of less experienced exercisers, the corresponding proportions were 7%, 25%, and 68% respectively.

The participants were also asked what kind of enjoyment they would sacrifice for one extra hour's exercise a week; 90% of the more experienced exercisers and 95% of the less experienced exercisers stated that they would give up watching TV, videos, and other such media.

Among more experienced exercisers, the measured costs of the time spent on exercise came to 7% of net wages; the corresponding figure among less experienced exercisers was 26% of net wages (p < 0.001). Most of the participants rated experience of exercise as high as or higher than experience of the leisure activity forgone. See Table 2.

**Table 2:** The costs of time spent on exercise expressed in percentage of wages, rated experience of exercise in relation to the leisure activity forgone and work, and ratings of the experience of the leisure activity foregone, work, and exercise on a scale from 0 (purely negative) to 100 (purely positive)

	More experienced exercisers	Less experienced exercisers	P-value
Number	164	41	
Cost of time (percentage of wages)	7.2%	26.3%	< 0.001
Rated experience of exercise the same as or higher than the leisure activity forgone (percentage of participants)	91.4%	72.5%	
Rated experience of exercise the same as or lower than work (percentage of participants)	5.5%	22.5%	
Rated experience of exercise between the leisure activity forgone and work (percentage of participants)	3.1%	5.5%	
Rating of the experience of leisure activity foregone	70.3	69.1	0.74
Rating of the experience of work	75.1	68.7	< 0.05
Rating of the experience of exercise	87.2	71.5	< 0.001

When the two study groups were merged, the cost of the time spent on exercise was correlated with a more positive experience of lost leisure activity (r = 0.25, p < 0.001) and a less positive experience of exercise (r = -0.45, p < 0.001). However, there was no significant correlation with positive experience of work (r = 0.00, p = 0.96). Hence, higher time costs can be explained by both a less positive experience of exercise and a more positive experience of the lost leisure activity.

Furthermore, the cost of time was correlated with the frequency (r = -0.18, p < 0.01) and duration of regular exercise (r = -0.18, p < 0.01).

## Discussion

### Principal findings

A model for measuring the costs of the time spent on exercise was proposed and tested in two groups of exercisers. According to the model, the costs of the time spent on exercise were significantly higher among less experienced exercisers than more experienced exercisers; this was due to a less positive experience of exercise and a more positive experience of the lost leisure activity. To our knowledge, this is the first attempt to measure the costs of the time spent on exercise, and the model seems to be an improvement over the arbitrary assumptions used in earlier economic evaluations.

Work time (on the margin) was assumed to represent a loss of utility in use compensated by net wages, and leisure activity (on the margin) to represent no loss of utility in use. The reality is less simple. Some of the participants seemed to enjoy their work, and some of the participants enjoyed their leisure activity (on the margin) less than their work. Two situations dominated the valuation. In the first, the experience of exercise was valued more highly than the experience of both lost enjoyment activity and work, and hence no net cost was accounted. In the second, the experience of exercise was valued less highly than the experience of both lost enjoyment activity and work; in this case, exercise time was accounted as costs equivalent to net wages. In the group of more experienced exercisers, the experience of exercise was valued more highly than the lost experience of enjoyment activity (87 vs. 70, p < 0.001), so these individuals in fact have benefits in utility in use besides the utility in anticipation.

### Strengths and weaknesses of the study

The model delivers the costs of time spent on exercise as valued by the participants. The questionnaire is easy to administer, and can be used to evaluate interventions in large study groups.

A critical part of the model is the comparison with the activity forgone. This activity needs to be the least valuable leisure time activity and motivated only by utility in use; while the activity may include some utility in anticipation, this utility must not motivate the activity. Thus, the utility in use of this activity represents the marginal value of leisure time. The most common activity forgone was watching television, videos, and other such media, an activity selected by more than 90% of the participants. While this activity might have some informational or knowledge value, the degree of utility in anticipation may be low and is rarely a motivator of the activity. Hence, the utility in use of the activity forgone in the investigation may represent the marginal value of leisure time.

The yardstick method with two anchoring points - utility of use of work and leisure activity forgone in favor of exercise - can only measure valuations between these two points. In fact, utility in use for exercise can be lower than that for work or higher than that for leisure activity forgone, but the method cannot measure how much lower or higher. In these cases, we have made the conservative assumption that the value is the same as for these two points. For the participants in the pilot study, this may have led to an underestimation of the value of exercise time, and hence an overestimation of the costs of exercise time.

In one way or another, our experience of something is always influenced by utility in anticipation. When we know that something is good for our health, we generally enjoy the activity more. The utility in anticipation will increase the utility in use, but the utility in anticipation per se (i.e. the expected health gain) is not accounted for in our model for valuation of time.

### Strengths and weaknesses in relation to other studies

Hatziandreu was the first to discuss the costs of the time spent on exercise, and her perspective has influenced later studies. She assumed that time costs were equivalent to net wages for those who disliked exercise, half net wages for those who were neutral, and zero for those who enjoyed exercise [8]. The costs of exercise time for the less experienced exercisers in our pilot study were around a quarter of net wages, which is generally somewhat lower than the assumptions made in previous studies. Our (small and non-representative) study indicates that the long-term costs may be much lower than these assumptions, and so valuations based on Hatziandreu's assumption may require rethinking. In reality, people do not always enjoy their lost leisure activity, and hence do not always need to enjoy exercise in order not to claim any utility in anticipation for taking exercise. On the other hand, people do not dislike their work, and hence do not need to dislike exercise in order to claim utility in anticipation for taking exercise at the level of net wages. This means that the gap between claiming net wages and not claiming any net wages seems to be closer than Hatziandreu supposed.

### Implications of the study

In cost-effectiveness analysis with a societal perspective, it is important to consider the costs of the time spent on exercise, particularly when methods of treating medical problems with medicine or other therapies are compared to methods based on patient time, such as promotion of physical activity.

### Unanswered questions and future research

This is the first attempt to measure the costs of the time spent on exercise, and should of course not be seen as the final solution. The next step is to use the model and questionnaire in an economic analysis of the promotion of physical activity. Experience from this practical use may open up the possibility of developing the method further.

## Conclusions

We have used the most important theoretical frameworks as a basis for developing a model and questionnaire for valuing time spent on exercise. The costs of this time were significantly higher among less experienced exercisers than more experienced exercisers; this was due to a less positive experience of exercise and a more positive experience of the lost leisure activity. In both groups, the time costs were lower than would be indicated by the existing rules of thumb.

Economic evaluations are mostly recommended to have a societal perspective and include all types of resources. Hence, the cost of time spent on exercise is an important factor in economic evaluations of interventions aimed at promoting physical activity.

This is the first attempt to find a method for measuring the time costs of exercise. Our method may produce better knowledge of time costs than previously-used assumptions which were not based on empirical data.

## Competing interests

The authors declare that they have no competing interests.

## Authors' contributions

LAH conducted the investigation, performed the statistical analysis, and drafted and revised the manuscript. LAH and LL together developed the model and questionnaire, and planned and designed the study. LL made revisions to the manuscript. Both authors have read and approved the final manuscript.
